# Neuronal Polarity: Positive and Negative Feedback Signals

**DOI:** 10.3389/fcell.2019.00069

**Published:** 2019-04-24

**Authors:** Tetsuya Takano, Yasuhiro Funahashi, Kozo Kaibuchi

**Affiliations:** ^1^Department of Cell Pharmacology, Nagoya University Graduate School of Medicine, Nagoya, Japan; ^2^Department of Cell Biology, Duke University Medical School, Durham, NC, United States

**Keywords:** neuronal polarity, axon specification, dendrite specification, positive feedback loop, negative feedback signal

## Abstract

Establishment and maintenance of neuronal polarity are critical for neuronal development and function. One of the fundamental questions in neurodevelopment is how neurons generate only one axon and several dendrites from multiple minor neurites. Over the past few decades, molecular and cell biological approaches have unveiled a large number of signaling networks regulating neuronal polarity in cultured hippocampal neurons and the developing cortex. Emerging evidence reveals that positive and negative feedback signals play a crucial role in axon and dendrite specification. Positive feedback signals are continuously activated in one of minor neurites and result in axon specification and elongation, whereas negative feedback signals are propagated from a nascent axon terminal to all minor neurites and inhibit the formation of multiple axon, thereby leading to dendrite specification, and maintaining neuronal polarity. This current insight provides a holistic picture of the signaling mechanisms underlying neuronal polarization during neuronal development. Here, our review highlights recent advancements in this fascinating field, with a focus on the positive, and negative feedback signals as key regulatory mechanisms underlying neuronal polarization.

## Introduction

Cell polarization is a crucial step in multiple cellular aspects such as differentiation, morphogenesis, and migration. Among the various cell types, neurons are highly polarized cells that have a long axon, and several short dendrites. These two different processes generate the information flow that is essential for brain functions such as memory, learning, and emotion. An axon is a single long process that transmits the information to other neurons by the release of neurotransmitters. Dendrites have several branched processes and dendritic spines, which contain neurotransmitter receptors to receive information from other neurons (Arimura and Kaibuchi, 2007; Takano et al., 2015). Recently, several excellent reviews have described remarkable advancements in understanding the molecular mechanisms regulating neuronal polarization, especially focusing on axon formation, and elongation *in vitro* and *in vivo* (Namba et al., 2015; Takano et al., 2015; Bentley and Banker, 2016; Schelski and Bradke, 2017; Yogev and Shen, 2017; Tortosa and Hoogenraad, 2018). In addition to these exciting topics, a major goal of neuronal development is to uncover the molecular mechanisms on how neurons stochastically determine axonal and dendritic fates to establish proper brain circuitry. Accumulating evidence has demonstrated that positive and negative feedback signals play a pivotal role in the establishment and maintenance of neuronal polarity (Arimura and Kaibuchi, 2007; Takano et al., 2015). These fascinating concepts can greatly improve the current understanding of signaling mechanisms regulating neuronal polarization. Moreover, recent studies suggest that both neuronal polarization and neuronal migration share common molecular mechanisms during neuronal development. Indeed, defects in neuronal polarization are closely tied to neuronal migration deficits in the developing cortex that result in neurodevelopmental disorders (Reiner and Sapir, 2009; Namba et al., 2015). In this brief review, we summarize the positive and negative feedback signals that are responsible for determining axonal and dendritic fates during neuronal development.

## Neuronal Polarization Processes

**FIGURE 1:**
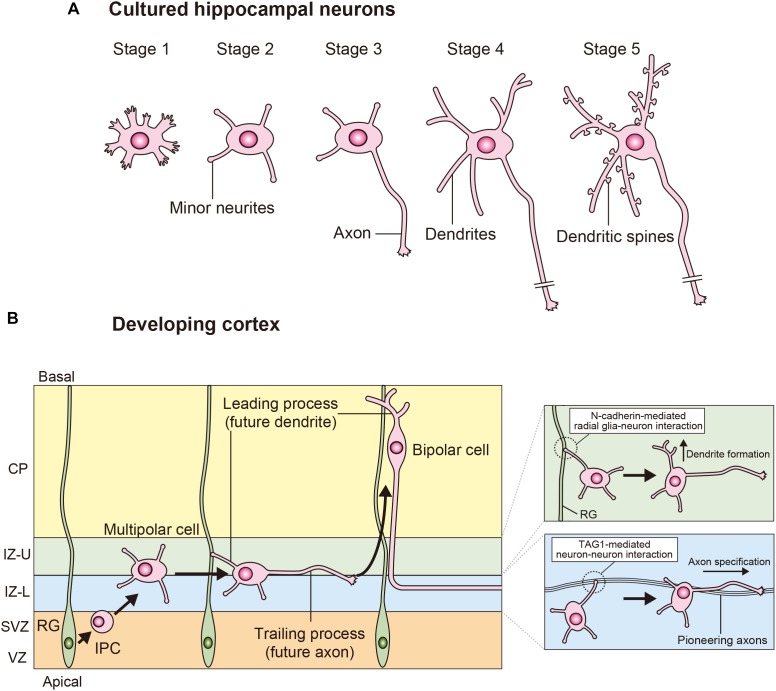
Processes of neuronal polarization *in vitro* and *in vivo*. **(A)** Upon isolation, hippocampal neurons form filopodia (stage 1). These neurons subsequently extend several minor neurites (stage 2), which show characteristic alternations of growth, and retraction. The major polarity event occurs when one of these equivalent minor neurites grows rapidly to become the axon (stage 3). The next steps are the morphological development of the remaining short minor neurites into dendrites (stage 4) and the functional polarization of axons and dendrites, including dendritic spine formation (stage 5). **(B)** In the developing neocortex, cortical neurons are generated from the radial glia in the VZ. These neurons extend multiple minor neurites and migrate toward the IZ through the SVZ. The multipolar (MP) cells generate a trailing process (future axon) and a leading process (future dendrite), and then these MP cells transform into bipolar (BP) cells and migrate toward the cortical plate (CP). A TAG-mediated interaction between a minor neurite of an MP cell and pioneering axons determines axon specification in the lower part of the IZ (IZ-L). N-cadherin-mediated interactions between the radial glia and neuron interactions determines dendrite specification in the upper part of the IZ (IZ-U).

Cultured hippocampal neurons have been an important experimental model for study neuronal polarity (Dotti et al., 1988; Banker, 2018). The neuronal morphological changes are classified into five stages (Figure 1A). Newly plated spherical hippocampal neurons extend filopodia (stage 1; shortly after plating). These neurons extend multiple minor neurites (stage 2; day 0.5–1.5), which are initially equivalent and undergo elongation and retraction. One of these equivalent minor neurites rapidly grows to become the axon (stage 3; day 1.5–3), and these neurons establish their polarity. The remaining short minor neurites continue to undergo growth and retraction, and these minor neurites subsequently develop into dendrites (stage 4; day 4–7). These neurons finally form dendritic spines and establish synaptic contacts (stage 5; >7 days in culture). Since axonal fate is stochastically determined in cultured hippocampal neurons, this process is called “the stochastic model” of neuronal polarization.

In the developing cortex, cortical neurons are generated in the ventricular zone (VZ) and subsequently form multiple minor neurites. These multipolar (MP) cells migrate toward the intermediate zone (IZ) through the subventricular zone (SVZ) (Miyata et al., 2004; Noctor et al., 2004). MP cells form a trailing process (future axon) and a leading process (future dendrite), and transform into bipolar (BP) cells in the IZ (Miyata et al., 2004; Noctor et al., 2004). BP cells migrate toward the cortical plate (CP) along radial glias. Since the formation of leading process is necessary for neuronal migration, the MP-to-BP transition is a crucial step not only in the establishment of neuronal polarity but also in neuronal migration in developing cortex (Figure 1B).

## Extrinsic Signaling in Axon and Dendrite Specification

**FIGURE 2:**
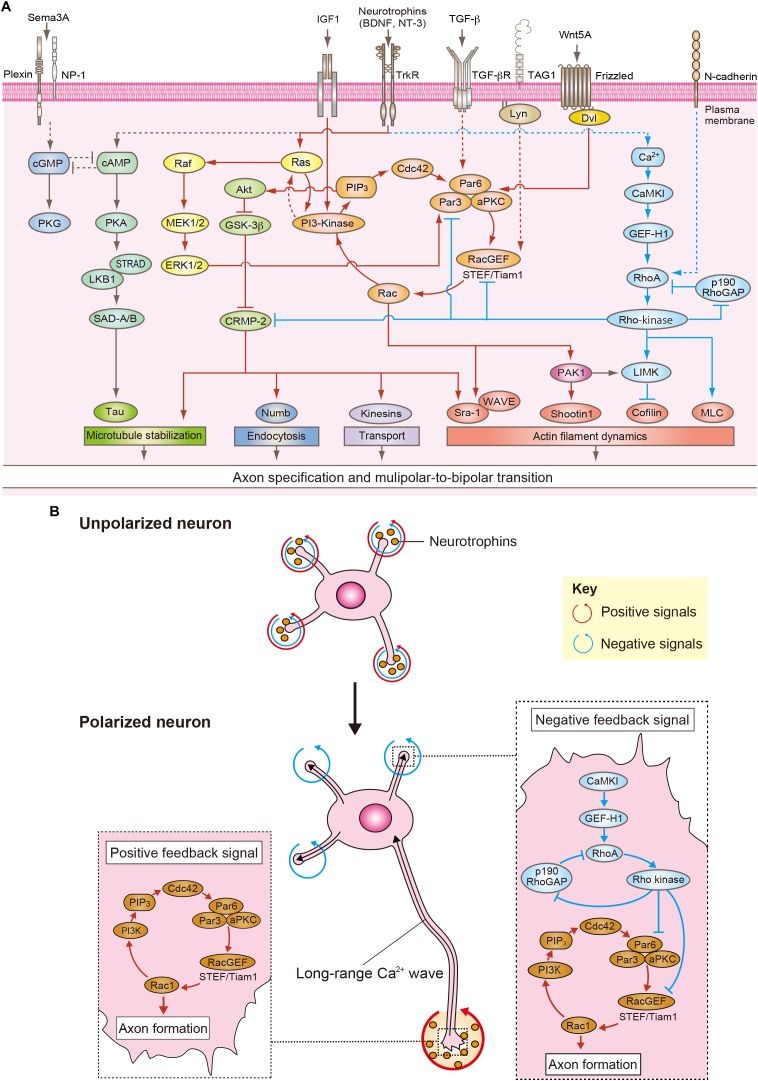
Positive and negative feedback signals for neuronal polarization. **(A)** Overview of positive (red arrows) and negative (blue arrows) feedback signaling that is responsible for neuronal polarization. Neurotrophins, TGF-β, Wnt5A, and TAG1 activate the Rac1/PI3-kinase/PIP_3_/Cdc42/Par complex/RacGEFs (STEF/Tiam) pathway that acts as a positive feedback loop underlying axon specification and MP-to-BP transition (red arrows). Neurotrophins induce Ras activation and thereby activate PI3-kinase. Ras also activates ERK1/2 through Raf/MEK pathway, and ERK1/2 regulates the selective transport of the Par complex to the nascent axon. TGF-β induces the phosphorylation of Par6, which is required for axon formation. Wnt5A activates aPKC through Dvl and promotes axon specification. TAG1 induces Rac1 activation through the Src family kinase Lyn. Rac1 regulates actin polymerization through PAK1-mediated phosphorylation of Shootin 1 and the Sra-1/WAVE complex during neuronal polarization. Rac1/PI3-kinase/PIP_3_/Cdc42/Par complex/RacGEFs (STEF/Tiam) pathway also activates CRMP-2 through Akt/GSK-3β pathway. CRMP-2 modulates microtubule stabilization, endocytosis, selective transport and actin filament dynamics through its binding to tubulin, Numb, the kinesin light chain subunit of kinesin-1, and Sra-1. Neurotrophins activate cAMP/PKA/LKB1/SAD-A/B kinases pathway, which regulates microtubule dynamics through the phosphorylation of Tau. Neurotrophins also induce Ca^2+^/CaMKK/CaMKI/GEF-H1/RhoA/Rho-kinase pathway that acts as a negative feedback signaling for preventing multiple axonal formation (blue arrows). N-cadherin activates RhoA/Rho-kinase. Rho-kinase maintains RhoA activation through inactivation of p190RhoGAP. Rho-kinase regulates actin filament dynamics and microtubule stabilization through MLC, LIM-kinase, and CRMP-2. Rho-kinase can inactivate Rac1 through the disruption of the Par complex and inactivation of Rac GEF (STEF). Semaphorin3A (Sema3A) suppresses axon formation through cGMP/PKG pathway. **(B)** In unpolarized neurons, the positive, and negative feedback signals are balanced in all minor neurites (red and blue arrows). When the balance is upset by the local amplification of neurotrophins, the Rac1/PI3-kinase/PIP_3_/Cdc42/Par complex/RacGEFs pathway is continuously activated within one minor neurite and thereby induces axon specification and elongation (red arrows). Concurrently, the long-range Ca^2+^ waves are propagated from the nascent axon to the cell body. The Ca^2+^ waves activate the CaMKI/GEF-H1/RhoA/Rho-kinase pathway, which represses the positive feedback loop in all minor neurites to prevent the formation of multiple axons and determine dendritic specification (blue arrows).

In the developing cortex, neurons are surrounded by specific microenvironments containing extracellular molecules such as neurotrophins [brain-derived neurotrophic factor (BDNF) and neurotrophin 3 (NT3)], insulin-like growth factor 1 (IGF1), Wnt5A, transforming growth factor-β (TGF-β) and Semaphorin 3A, and cell adhesion molecules such as TAG1 and N-cadherin, which provide cues for neuronal polarization (Figure 2A; Funahashi et al., 2014; Namba et al., 2015; Takano et al., 2015; Rozes-Salvador et al., 2018). Indeed, inhibition of neurotrophin receptors, TrkB and TrkC, block the MP-to-BP transition *in vivo* (Nakamuta et al., 2011). Knockdown of TrkB also shows impairment of neuronal migration (Cheng et al., 2011). Recently, it has been shown that knockdown of the IGF-1 receptor impairs the MP-to-BP transition and neuronal migration (Nieto Guil et al., 2017). The expression level of Wnt5A is increased during the MP-to-BP transition in IZ and inhibition of Wnt5A blocks the MP-to-BP transition and neuronal migration (Boitard et al., 2015). Wnt5A activates atypical PKC (aPKC) in complex with Par3 and Par6 through Disheveled (Dvl) and promotes axon specification (Zhang et al., 2007). Because TGF-β is highly expressed in the VZ compared to that of CP in the developing cortex, the expression pattern of TGF-β is graded along the VZ-to-CP axis. TGF-β receptor (TβR2) conditional knockout mice fail to form axons of pyramidal neurons *in vivo* (Yi et al., 2010). TβR2 induces axon formation through phosphorylation of Par6 (Yi et al., 2010). In contrast, Semaphorin 3A is predominantly expressed in the CP and its expression decreases in the VZ (Polleux et al., 2000). Semaphorin 3A suppresses axon formation and promotes dendrite formation *in vitro*, and regulates the MP-to-BP transition and neuronal migration *in vivo* (Shelly et al., 2011). The gradient of these secreted factors was initially thought to determine axon or dendrite specification (Polleux et al., 2000; Yi et al., 2010). However, recent studies have shown that MP cells form the trailing process in any direction and subsequently migrate toward the CP, leaving behind the trailing process, which results in axonal elongation toward the VZ (Nakamuta et al., 2011; Namba et al., 2014). A gradient of extracellular molecules might be responsible for the axon formation and directional migration, but not for the determination of axon specification.

Given that MP cells constantly establish neuronal polarity within the IZ, cell-to-cell interactions should be noted. In the lower part of the IZ, approximately 60% of the MP cells first generate a trailing process and subsequently form a leading process (Hatanaka and Yamauchi, 2013; Takano et al., 2015). In this region. pioneering axons of cortical neurons are enriched (Namba et al., 2014). When a minor neurite of an MP cell “touches” a pioneering axon, it rapidly extends (“goes”) and becomes an axon. This process has been proposed the “Touch & Go model” (Namba et al., 2014, 2015; Takano et al., 2015). The cell adhesion molecule TAG1 is highly expressed in the pioneering axons and regulates this process. The TAG1-mediated neuron-neuron interaction activates Rac1 at the minor neurite of the MP cell through the Src family kinase Lyn and induces axon initiation (Namba et al., 2014; Figure 2A). Thus, TAG1-mediated neuron-neuron interaction in the lower part of IZ is essential for axon specification in the developing cortex (Namba et al., 2014; Figure 1B). The remaining approximately 40% of MP cells first form the leading process and then generate the trailing process in the upper part of IZ (Namba et al., 2014; Takano et al., 2015). In this region, a minor neurite of an MP cell becomes the leading process by making contact with a radial glia (Xu et al., 2015). N-cadherin accumulates in the upper part of IZ and regulates the radial glia-neuron interactions that determine neuronal polarity (Xu et al., 2015; Figure 1B). Inhibition of N-cadherin by the expression of dominant-negative N-cadherin or knockdown disrupts axon-dendrite polarity and neuronal migration *in vivo* (Kawauchi et al., 2010; Jossin and Cooper, 2011; Gartner et al., 2012; Xu et al., 2015). N-cadherin activates RhoA at the contacting neurite and then induces the formation of a leading process (Xu et al., 2015; Figure 2A). Thus, the TAG1-mediated neuron-neuron interaction and the N-cadherin-mediated radial glia–neuron interaction are essential for neuronal polarization *in vivo* as complementary mechanisms (Figure 1B).

## Positive Feedback Signals

The involvement of “positive and negative feedback signals” play a crucial role in neuronal polarization (Arimura and Kaibuchi, 2007; Inagaki et al., 2011; Takano et al., 2015; Schelski and Bradke, 2017). “Positive feedback signals” induce axon specification and elongation. “Negative feedback signals” determine dendrite specification and maintain neuronal polarity by preventing the formation of multiple axons.

In the developing cortex, neuronal polarization is established in response to environmental cues (Funahashi et al., 2014; Namba et al., 2015). In contrast, cultured neurons stochastically generate a single axon and multiple dendrites that never develop into axons without the need for exogenous factors (Arimura and Kaibuchi, 2007; Takano et al., 2015). How do neurons on the stochastic model establish their polarity without these environmental cues? Recent results showed that inhibition of neurotrophins, such as BDNF and NT-3, or their receptors through the use of neutralizing antibodies and inhibitors prevents axon specification. These findings indicate that cultured hippocampal neurons secrete these neurotrophins for establishing neuronal polarity (Cheng et al., 2011; Nakamuta et al., 2011). Neurotrophin receptors are predominantly transported toward the tip of the nascent axon by kinesin-1 (Arimura et al., 2009). Furthermore, BDNF induces the production of cAMP and activation of PKA, which leads to further BDNF secretion and membrane insertion of TrkB, indicating that local amplification of neurotrophin signals are essential for neuronal polarity (Arimura et al., 2009; Cheng et al., 2011; Nakamuta et al., 2011).

The local amplification of neurotrophins induces PKA-dependent phophsoryaltion of the serine/threonine kinase LKB1, which promotes axon specification (Barnes et al., 2007; Shelly et al., 2007). LKB1 interacts with the STE20-related pseudokinase (STRAD) and the LKB1/STRAD complex is enriched in the growing axon (Shelly et al., 2007). Overexpression of LKB1 induces the formation of multiple axons, whereas inhibition of LKB1 attenuates axon specification in cultured hippocampal neurons (Barnes et al., 2007; Shelly et al., 2007). LKB1 conditional knockout mice fail to form axons of pyramidal neurons in the cortex, but neuronal migration is unaffected (Barnes et al., 2007). LKB1 phosphorylates and activates SAD-A/-B kinases, which regulate microtubule dynamics through the phosphorylation of Tau during neuronal polarization (Barnes et al., 2007; Shelly and Poo, 2011). SAD-A/-B kinases-deficient neurons show a mixed axon/dendrite identity in the developing neocortex (Kishi et al., 2005).

Neurotrophins also activate phosphoinositide 3-kinase (PI3-kinase) by Ras activation, thereby leading to axon specification *in vitro* (Shi et al., 2003; Menager et al., 2004; Yoshimura et al., 2006). Ras activation is abolished by the action of the PI3-kinase inhibitor in the tip of the axon, indicating a positive feedback loop between Ras, and PI3-kinase appears to be involved in neuronal polarization (Fivaz et al., 2008; Figure 2A). However, the molecular mechanism of Ras activation by PI3-kinase that is responsible for axon specification remains unknown. PI3-kinase induces activation of Cdc42 by phosphatidylinositol 3,4,5-trisphosphate (PIP_3_) (Menager et al., 2004; Nishimura et al., 2005; Reichardt, 2006). Cdc42 interacts with the Par complex and then induces Rac1 activation through Rac-specific GEFs (STEF/Tiam1). The active Rac1 leads to further activation of PI3-kinase (Nishimura et al., 2005). Thus, the PI3-kinase/Cdc42/Par complex/RacGEF/Rac1 pathway represents a positive-feedback loop that responsible for axon specification (Arimura and Kaibuchi, 2007; Takano et al., 2015; Figure 2B). The Par complex is selectively transported into the nascent axon through the interaction of KIF3A (Nishimura et al., 2004). A recent study showed that this interaction is regulated by extracellular signal-regulated kinase 2 (ERK2)-mediated phosphorylation of Par3 and is important for neuronal polarization *in vivo* (Funahashi et al., 2013). In the developing cortex, the constitutively active or dominant-negative form of Rac1 or STEF/Tiam1 shows a loss of leading and trailing processes and neuronal migration defects, indicating that the appropriate activity of Rac1 is essential for MP-to-BP transition and neuronal migration (Kawauchi et al., 2003). Moreover, knockdown of Par3 disrupts the MP-to-BP transition and neuronal migration (Funahashi et al., 2013). These findings suggest that the PI3-kinase/Cdc42/Par complex/RacGEF/Rac1 pathway has a vital role in the establishment of neuronal polarization as a positive feedback signal.

## Downstream Targets of Positive Feedback Signals

How do positive feedback signals regulate axon specification and elongation? Positive feedback signals lead to a local accumulation of additional effectors such as CRMP-2, Par complex, and Shootin1 in the nascent axon during neuronal polarization (Toriyama et al., 2010; Inagaki et al., 2011; Naoki et al., 2011; Naoki and Ishii, 2014). These accumulated molecules govern multiple cellular events including microtubule stabilization, endocytosis, selective transport, and actin filament dynamics to induce axon specification and elongation (Conde and Caceres, 2009; Namba et al., 2015; Schelski and Bradke, 2017; Figure 2A).

The PI3-kinase/Cdc42/Par complex/RacGEF/Rac1 pathway phosphorylates and inactivates glycogen synthase kinase-3β (GSK-3β) through interleukin-like kinase (ILK) and Akt. Phosphorylated GSK-3β is enriched in the nascent axon (Jiang et al., 2005; Yoshimura et al., 2005). The expression of a constitutive active mutant form of GSK-3β inhibits axon specification, whereas inhibition of GSK-3β induces multiple axonal formation (Jiang et al., 2005; Yoshimura et al., 2005). GSK-3β phosphorylates CRMP-2 at Thr514 and suppresses its activity (Yoshimura et al., 2005). The expression of CRMP-2 induces the formation of multiple axons in culred hippocampal neurons (Inagaki et al., 2001). Consistently, inhibition of CRMP-2 suppresses axon formation *in vitro* and MP-to-BP transition *in vivo* (Inagaki et al., 2001; Sun et al., 2010). CRMP-2 regulates microtubule dynamics that are essential for axon specification (Arimura and Kaibuchi, 2007; Witte and Bradke, 2008; Takano et al., 2015; Schelski and Bradke, 2017). Indeed, the microtubule stabilizing agent Taxol induces the formation of multiple axons *in vitro*, indicating that the microtubule stabilization is required for axon specification (Witte and Bradke, 2008; Schelski and Bradke, 2017). CRMP-2 also modulates endocytosis, selective transport and actin filament dynamics through its binding to Numb, the kinesin light chain subunit of kinesin-1 and Sra-1 (Arimura and Kaibuchi, 2007; Takano et al., 2015; Figure 2A).

The Par complex (Par3/Par6/aPKC) was identified as a key regulator of cell polarity by a genetic screen for mutaions that disturb asymmetric cell divisions (Kemphues et al., 1988; Rodriguez-Boulan and Macara, 2014). The Par complex is enriched in the growing axon by KIF3A-dependent transport (Shi et al., 2003; Nishimura et al., 2004; Funahashi et al., 2013). The phosphorylation of Par3 at Ser1116 by ERK2 disrupts this Par3-KIF3A interaction and thereby leads to the accumulation of the Par complex at the nascent axon that results in axon specification (Funahashi et al., 2014). Knockdown of Par3 disrupts the MP-to-BP transition *in vivo* (Chen et al., 2013; Funahashi et al., 2013). The phospho-mimic mutant of Par3, which cannot bind to KIF3A, failed to rescue the knockdown phenotype, indicating that the accumulation of Par3 in the nascent axon is essential for MP-to-BP transition *in vivo* (Funahashi et al., 2013). Par6 also colocalizes with the TGF-β receptor (TβR2) and the phosphorylation of Par6 at Ser345 by the TGF-β receptor regulates axon formation and neuronal migration (Ozdamar et al., 2005; Yi et al., 2010; Rozes-Salvador et al., 2018).

Rac1 is a key regulator of actin polymerization during neuronal polarization through p21 activated kinase (PAK) and the Sra/Wiskott–Aldrich syndrome protein (WASP)-family verprolin-homologs protein (WAVE) complex (Kawano et al., 2005; Tahirovic et al., 2010; Schelski and Bradke, 2017). PAK1 phosphorylates Shootin1, which is selectively transported in a neurite-length-dependent manner and leads to axon specification in cultured hippocampal neurons (Toriyama et al., 2006; Toriyama et al., 2010). Phosphorylation of Shootin1 mediates binding between L1 cell adhesion molecule (L1-CAM) and F-actin retrograde flow as a molecular clutch (Toriyama et al., 2013; Kubo et al., 2015). This interaction generates a traction force in the growth cone of the nascent axon through decreasing retrograde actin flow, thereby increasing actin polymerization for pushing against the membrane of the nascent axon. In the developing cortex, knockdown of Shootin1 impairs the MP-to-BP transition, and neuronal migration (Sapir et al., 2013). Rac1 also recruits the WAVE complex to the plasma membrane of the nascent axon, and the WAVE complex regulates actin filament dynamics through actin related protein 2/3 (Arp2/3) to induce axon elongation (Tahirovic et al., 2010; San Miguel-Ruiz and Letourneau, 2014). Thus, actin filaments are more dynamic in the nascent axon than in those of other minor neurites (Witte and Bradke, 2008; Schelski and Bradke, 2017). Indeed, actin destabilizing drugs induce the formation of multiple axons, and local application of the actin destabilizing drugs to one minor neurite leads to axon specification (Bradke and Dotti, 1999; Witte and Bradke, 2008). These results indicate that actin filament dynamics are required for determining axonal specification. Altogether, positive feedback signals are continuously activated within one minor neurite and result in axon specification through many downstream pathways such as cytoskeletal organization and intracellular trafficking (Arimura and Kaibuchi, 2007; Takano et al., 2015; Figure 2A).

## Negative Feedback Signals

Since neurons constantly generate only one axon and other minor neurites that never become axons, negative feedback signals play an important role in the formation of a single axon, and multiple dendrites during neuronal development (Arimura and Kaibuchi, 2007; Takano et al., 2015). Several models of negative feedback signals have been proposed (Inagaki et al., 2011; Takano et al., 2015; Schelski and Bradke, 2017; Yogev and Shen, 2017). cAMP and cGMP show antagonistic actions on each other during neuronal polarization (Shelly et al., 2010). Local elevation of cAMP level induces axon specification through PKA activation, whereas the cGMP level is increased by reducing the amount of cAMP in other minor neurites and then leads to dendritic specification (Shelly et al., 2010). However, it remains unclear how the cAMP elevation in the nascent axon regulates the cGMP level in all of the other minor neurites. It has also been proposed that there is a winner-take-all model for the establishment of neuronal polarity (Inagaki et al., 2011; Schelski and Bradke, 2017). As the amount of growth-relating factors are limited, local accumulation of these factors in the nascent axon deplete them in all of the other minor neurites. In turn, all of the other minor neurites could not become axons (Inagaki et al., 2011; Schelski and Bradke, 2017). More recently, a spatiotemporal long-range negative feedback signal for guaranteeing the proper neuronal polarization has been identified (Takano et al., 2017). The negative feedback signal is mediated by unique long-range Ca^2+^ waves, which are generated by NT-3 and propagate from the growing nascent axon to the cell body (Takano et al., 2017). The long-range Ca^2+^ waves subsequently activate calmodulin-dependent protein kinase I (CaMKI) at the cell body (Takano et al., 2017). CaMKI induces phosphorylation and activation of a RhoA-specific GEF, GEF-H1, and thereby activates RhoA and its effector Rho-kinase at the cell body (Takano et al., 2017). RhoA and Rho-kinase are well known as key negative regulators of neurogenesis through modulating the actin cytoskeleton and myosin-based contractility in several cell lines (Da Silva et al., 2003; Conde et al., 2010). Interestingly, photoactivation of RhoA or Rho-kinase by an optogenetic approach, LOV2 trap and release of the protein (LOVTRAP), in the cell body specifically inhibits minor neurite elongation in polarized neurons (Takano et al., 2017). Additionally, computational modeling has shown that active Rho-kinase spreads from the cell body into the minor neurites but not into the axons for preventing multiple axonal formation (Takano et al., 2017). Consistently, inhibition of Rho-kinase induces minor neurite elongation that develops into multiple axons (Takano et al., 2017). These results indicate the polarized activation of RhoA/Rho-kinase is necessary for generation of the single axon and multiple dendrites during neuronal development (Gonzalez-Billault et al., 2012; Takano et al., 2017). In the developing cortex, inhibition of RhoA or Rho-kinase by the expression of the dominant negative mutant impairs the MP-to-BP transition, and neuronal migration (Xu et al., 2015). The expression of a phospho-mimic mutant of GEF-H1, which leads to RhoA activation, also disrupts the MP-to-BP transition and neuronal migration, indicating the polarized RhoA/Rho-kinase activity is essential for neuronal polarization and neuronal migration *in vivo* (Xu et al., 2015; Takano et al., 2017). Rho-kinase maintains RhoA activation through phosphorylation and inactivation of p190RhoGAP (Mori et al., 2009). Importantly, Rho-kinase can inactivate Rac1 through the disruption of Par complex and inactivation of STEF in a phosphorylation-dependent manner (Takefuji et al., 2007; Nakayama et al., 2008). Thus, the long-range Ca^2+^ waves/CaMKI/GEF-H1/RhoA/Rho-kinase pathway represents a negative feedback signal that functions to repress the positive feedback loop in all minor neurites to inhibit multiple axonal formation and determine dendritic specification (Arimura and Kaibuchi, 2007; Takano et al., 2015, 2017; Figure 2). Together, Rac1-dependent positive feedback signals in the nascent axon and RhoA/Rho-kinase-dependent negative feedback signals in all other minor neurites guarantee proper neuronal polarization (Figure 2B).

## Concluding Remarks

Banker and colleagues discovered neuronal morphological changes using cultured hippocampal neurons and reported it as “neuronal polarity” (Dotti et al., 1988; Banker, 2018). Over the past three decades, this new research field has been growing and revealed a large number of signaling networks regulating neuronal polarity (Arimura and Kaibuchi, 2007; Barnes and Polleux, 2009; Takano et al., 2015; Schelski and Bradke, 2017). Particularly important in this regard are most of the environmental polarity cues being tightly connected to Rac1-dependent positive feedback signals and RhoA/Rho-kinase-dependent negative feedback signlas (Figure 2). The positive and negative feedback signals remodel the actin and microtubule cytoskeleton underlying axon and dendrite specification during neuronal development. Rac1-depedent positive feedback signals induce dynamics of actin filaments and stabilization of microtubules in one of minor neurites through several downstream molecules including PAK1, the Sra-1/WAVE complex and CRMP-2, thereby leading to axon specification and elongation (Kawano et al., 2005; Tahirovic et al., 2010; Schelski and Bradke, 2017; Figure 2). In contrast, RhoA/Rho-kinase-dependent negative feedback signal stabilizes actin filaments in the minor neurites through myosin light chain (MLC) and LIM-kinase to prevent the formation of multiple axons and determine dendritic specification (Tahirovic and Bradke, 2009; Amano et al., 2010; Takano et al., 2017; Figure 2). Although negative feedback signals that are delivered from the nascent axon induce dendritic specification in cultured hippocampal neurons, some neurons that do not have axons by knockout of TGF-β receptor or LKB1 show dendrite formation in the developing cortex (Barnes et al., 2007; Yi et al., 2010). These findings suggest that additional molecular mechanisms might be involved in dendritic specification *in vivo*. Recently, it has been shown that N-cadherin-mediated glia-neuron interactions determine dendritic specification before axon formation (Takano et al., 2015; Xu et al., 2015). Interestingly, N-cadherin also activates RhoA/Rho-kinase in this process, indicating that RhoA/Rho-kinase plays critical role in dendritic specification during neuronal development. Thus, Rac1-dependent positive feedback signals and RhoA/Rho-kinase-dependent negative feedback signals are major key regulatory systems underlying neuronal polarization. However, our current knowledge of the signaling networks regulating neuronal polarization *in vivo* remains underdeveloped. In recent years, *in utero* electroporation approaches have become very useful for analyzing the role of various molecules on neuronal polarity in the mammalian cerebral cortex (Saito and Nakatsuji, 2001; Namba et al., 2014; Xu et al., 2015; Takano et al., 2017). However, it is difficult to distinguish the neuronal polarity defects and the neuronal migration deficits because neuronal polarization occurs during neuronal migration in the neocortex. Furthermore, these defects have a tremendous effect on the synaptic connections, which result in neurodevelopmental disorders such as schizophrenia, autism, lissencephaly, and microcephaly (Francis et al., 2006; Manzini and Walsh, 2011; Ishii et al., 2016; Reiner et al., 2016). How an altered neuronal polarization impacts these diseases has remained an open question. Future studies are necessary to uncover not only molecular mechanisms underlying neuronal polarization *in vivo* but also how these mechanisms are coordinated with other neuronal events including neuronal migration and synaptic functions to establish proper brain circuits. These broad approaches to study neuronal polarization would provide new insight into neuronal development and shed light on therapeutic approaches for neurodevelopmental disorders.

## Author Contributions

TT and KK wrote the manuscript. YF contributed to the discussion about the manuscript.

## Conflict of Interest Statement

The authors declare that the research was conducted in the absence of any commercial or financial relationships that could be construed as a potential conflict of interest.
